# Automated detection of MRI-negative temporal lobe epilepsy with ROI-based morphometric features and machine learning

**DOI:** 10.3389/fneur.2024.1323623

**Published:** 2024-01-31

**Authors:** Lin Yang, Bo Peng, Wei Gao, Rixi A, Yan Liu, Jiawei Liang, Mo Zhu, Haiyang Hu, Zuhong Lu, Chunying Pang, Yakang Dai, Yu Sun

**Affiliations:** ^1^School of Biological Science and Medical Engineering, Southeast University, Nanjing, China; ^2^Department of Radiology, The First Affiliated Hospital of Soochow University, Suzhou, China; ^3^Suzhou Institute of Biomedical Engineering, Chinese Academy of Sciences, Suzhou, China; ^4^Jinan Guoke Medical Engineering Technology Development Co., Ltd, Jinan, China; ^5^Department of Neurosurgery, The First Affiliated Hospital of Soochow University, Suzhou, China; ^6^School of Life Science and Technology, Changchun University of Science and Technology, Changchun, China; ^7^International Laboratory for Children’s Medical Imaging Research, School of Biological Science and Medical Engineering, Southeast University, Nanjing, China; ^8^Institute of Cancer and Genomic Sciences, University of Birmingham, Birmingham, United Kingdom

**Keywords:** magnetic resonance imaging, temporal lobe epilepsy, gray matter volume, cortical thickness, cortical surface area, machine learning

## Abstract

**Objective:**

Temporal lobe epilepsy (TLE) predominantly originates from the anteromedial basal region of the temporal lobe, and its prognosis is generally favorable following surgical intervention. However, TLE often appears negative in magnetic resonance imaging (MRI), making it difficult to quantitatively diagnose the condition solely based on clinical symptoms. There is a pressing need for a quantitative, automated method for detecting TLE.

**Methods:**

This study employed MRI scans and clinical data from 51 retrospective epilepsy cases, dividing them into two groups: 34 patients in TLE group and 17 patients in non-TLE group. The criteria for defining the TLE group were successful surgical removal of the epileptogenic zone in the temporal lobe and a favorable postoperative prognosis. A standard procedure was used for normalization, brain extraction, tissue segmentation, regional brain partitioning, and cortical reconstruction of T1 structural MRI images. Morphometric features such as gray matter volume, cortical thickness, and surface area were extracted from a total of 20 temporal lobe regions in both hemispheres. Support vector machine (SVM), extreme learning machine (ELM), and cmcRVFL+ classifiers were employed for model training and validated using 10-fold cross-validation.

**Results:**

The results demonstrated that employing ELM classifiers in conjunction with specific temporal lobe gray matter volume features led to a better identification of TLE. The classification accuracy was 92.79%, with an area under the curve (AUC) value of 0.8019.

**Conclusion:**

The method proposed in this study can significantly assist in the preoperative identification of TLE patients. By employing this method, TLE can be included in surgical criteria, which could alleviate patient symptoms and improve prognosis, thereby bearing substantial clinical significance.

## Introduction

Epilepsy is a clinical condition characterized by recurrent abnormal neuronal discharges, leading to various symptoms (1, 2). Temporal lobe epilepsy (TLE) mainly originates from the anteromedial basal region of the temporal lobe and often has a favorable prognosis following surgical intervention (3). The epidemiology of TLE involves factors such as age, gender, and potential causes. The incidence of TLE peaks in early childhood and later in adulthood, with some cases linked to head injuries, infections, or structural brain abnormalities (4). Identifying TLE patients preoperatively is crucial for tailoring treatment strategies, optimizing surgical outcomes, and ultimately improving the overall well-being of individuals with epilepsy. For precision preoperative planning, identification allows for precise localization of the epileptic focus with minimal impact on non-affected brain regions (5). For optimizing surgical outcomes, early identification aids in optimizing surgical outcomes by ensuring that the surgical procedure targets the specific area responsible for seizures, improving the likelihood of seizure control and reduce the risk of complications (6). For enhancing quality of life, timely identification and effective surgical management contribute to improved quality of life for individuals with TLE, reducing seizure frequency and better cognitive outcomes can positively impact daily functioning and overall well-being (7). Therefore, Identifying TLE patients preoperatively is an important issue in the clinical diagnosis and treatment of epilepsy.

Magnetic resonance imaging (MRI) has been extensively used for locating epileptogenic foci due to its high soft tissue resolution and absence of radiation exposure, making it a potent tool for studying structural abnormalities in epilepsy (8–11). Research has shown that MRI has significant diagnostic value for TLE; for instance, the amygdala volume is notably reduced in TLE patients (12), and some patients also present with low-grade gliomas and peripheral vascular lesions (13). MRI also aids in localizing abnormal brain tissues commonly found in TLE, such as cortical dysplasia and hippocampal sclerosis (8, 14). However, these structural changes are difficult to identify directly by visual inspection of MRI scans.

In recent years, machine learning methods have been widely applied in the classification and diagnosis of neuropsychiatric disorders. Support Vector Machine (SVM), Extreme Learning Machine (ELM), and Random Vector Functional Link (RVFL) are common classifiers to handle complex relationships in data and make predictions, which are widely applied into analysis of neuropsychiatric disorders. SVM is a popular supervised learning algorithm for classification and regression tasks (15). It aims to find a hyperplane that best separates data into different classes while maximizing the margin between them. SVMs are effective in high-dimensional spaces and are used for various applications, including image classification and bioinformatics. Huang et al. analyzed the increased network homogeneity in the TLE patients using support vector machine (SVM) with a classification accuracy of 74.12% (16). ELM is a type of neural network that simplifies the training process compared to traditional neural networks (17). It randomly selects input weights and analytically determines the output weights. Wang et al. used discrete wavelet transform and the nonlinear sparse ELM for epilepsy and epileptic seizure detection (18). RVFL is another type of neural network that combines randomization with a feedforward neural network structure (19). It randomly assigns input weights and biases, and the output weights are calculated through a least squares solution. Goel et al. used wavelet transform-based multimodality fusion and RVFL classifier to incorporate structural and metabolic information for the early detection of the neurodegenerative disease (20). While most studies have a TLE diagnosis rate exceeding 80%, approximately 10–20% of TLE patients are misdiagnosed, and a considerable proportion of healthy individuals are falsely identified, indicating the need for a more precise auxiliary diagnostic method for TLE.

In this study, we propose a TLE identification method based on morphological features of temporal lobe regions and machine learning techniques. By analyzing MRI scans of 32 retrospective epilepsy cases and using surgical removal of the epileptogenic zone in the temporal lobe with a good postoperative prognosis as the criteria for TLE, patients were classified into 20 TLE and 12 epilepsy originating from other regions. Morphological features were extracted from relevant temporal lobe regions, and several machine learning models were utilized to evaluate the morphological features as the neuroimaging diagnostic biomarkers for TLE. The aim is to develop an automated, quantitative, and highly accurate preoperative TLE identification method.

## Materials and methods

### Patients

This study included a cohort of 51 epilepsy patients, all of whom were recruited from the First Affiliated Hospital of Suzhou University (27 males and 24 females). The sample consisted of 34 patients diagnosed with temporal lobe epilepsy (TLE) and 17 with epilepsy originating from other brain regions (non-TLE). The inclusion and exclusion criteria for the epilepsy patients in this study were as follows: (1) The primary basis for the diagnosis of epilepsy was epileptiform discharges on electroencephalogram (EEG), coupled with clinical seizures and medical history. (2) Patients were excluded with absence of visible structural abnormalities or lesions on routine neuroimaging studies, that could account for the seizures. (3) Patients lacking preoperative T1-weighted images were excluded from the study. (4) Patients with MRI scans of poor quality were also excluded. The identification of the epileptogenic foci for the patients with epilepsy was determined through a combination of postoperative MRI scans and surgical records. The surgical records included details about the excised regions, such as the frontal lobe, occipital lobe, hippocampus, amygdala, middle and inferior temporal gyrus, superior temporal gyrus, and other typical areas. Demographic information and MRI lesion information of all patients is shown in Table 1.

**Table 1:** Demographic information and MRI lesion information of all epilepsy patients.

	TLE group	Non-TLE group	*p*-value
Participants	34	17	–
Sex (male/female, %)	Female: 16 (47%) Male: 18 (53%)	Female: 8 (47%) Male: 9 (53%)	0.50
Age (mean SD, years)	32.29 ± 35.71	25.59 ± 17.59	0.07
Hemisphere(left/right, %)	Left: 16 (47%) Right: 18 (53%)	Left: 6 (54.5%) Right: 11 (45.5%)	0.21
Lesion location (lobe, %)	Temporal lobe: 34 (100%)	Precentral gyrus: 5(%) Occipital lobe: 11.8 (%) Frontal lobe:29.4 (%) Parietal lobe and Occipital lobe: 52.9 (%)	–

TLE, temporal lobe epilepsy; SD, standard deviation.

### MR image acquisition

All participants underwent the clinical standard brain MRI protocol, including T1-weighted MRI, T2-weighted MRI, FLAIR, and DWI. The brain MR images were acquired at the First Affiliated Hospital of Suzhou University using a Siemens MAGNETOM Skyra 3.0 T ultraquiet MRI system and Philips MR Achieva/Intera equipment to obtain T1-weighted MRI scans.

For the Siemens system, the scanning parameters were as follows:

Repetition Time (TR)/Echo Time (TE) = 2300.0/3.0 ms.

Slice Thickness = 1.0 mm.

Flip Angle (FA) = 8°.

For the Philips medical equipment, the scanning parameters were as follows:

Repetition Time (TR)/Echo Time (TE) = 8.1/3.7 ms.

Slice Thickness = 1.0 mm.

Flip Angle (FA) = 9°.

### Image processing

**Figure 1:**
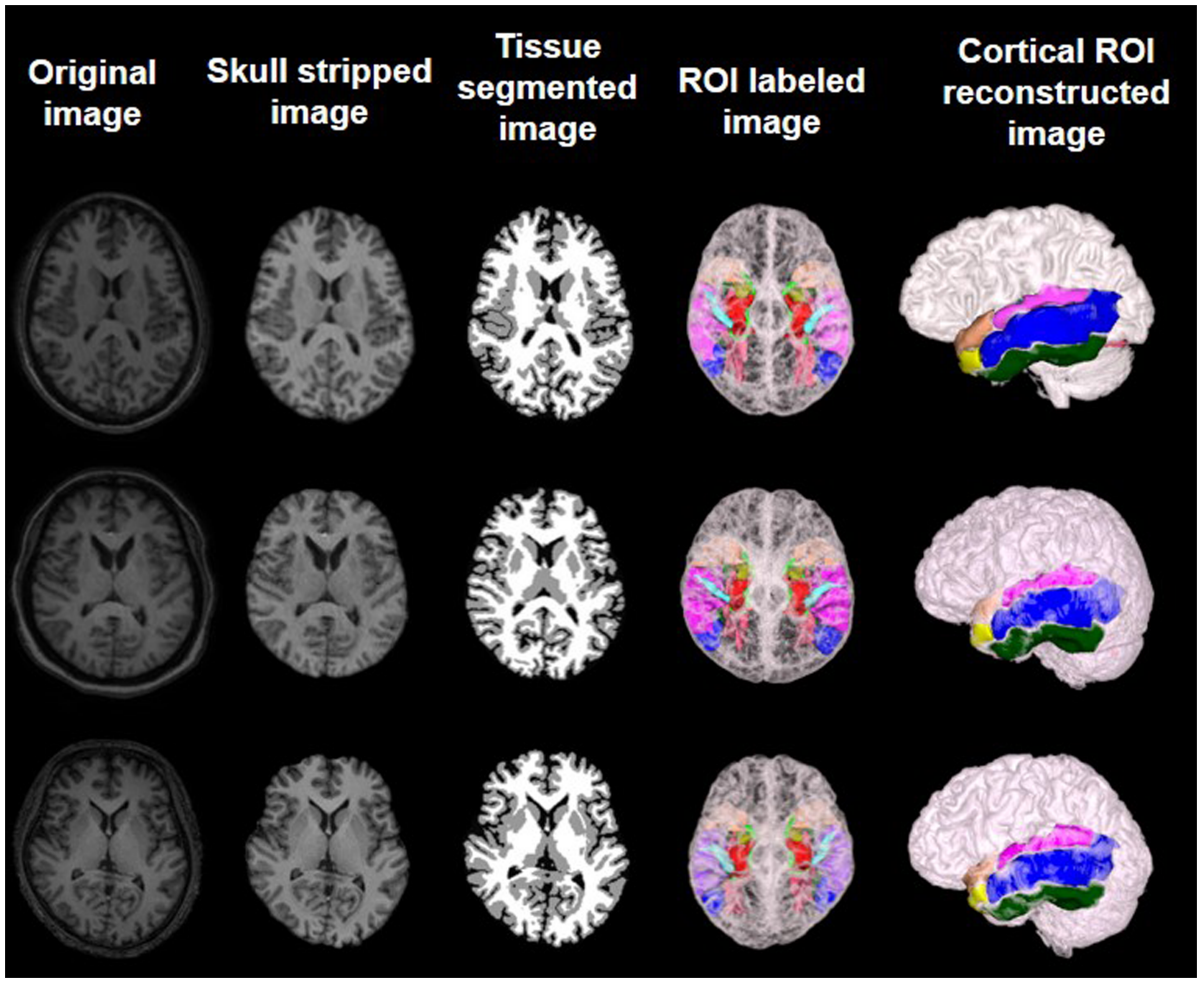
Preprocessed MR images and feature extraction on brain labeled and cortical reconstructed MR images of three different subjects.

MRI brain images were processed using standard protocols as outlined in previous literature (21). Because the brain MR images of all patients were derived from two different devices, several preprocessing steps are recommended for consistency, including intensity normalization and spatial normalization. Intensity normalization of MR images from both devices could mitigate differences in signal strength and ensure uniform grayscale representation. Spatial normalization could transform images into a common coordinate space, facilitating anatomical comparisons across subjects. After spatial normalization, the intensity of images was normalized to 0 ~ 250. After intensity normalization, the raw MRI images have uniform dimensions (256 × 256 × 256) and a consistent spatial resolution of 1 × 1 × 1 mm^3^. The N3 algorithm was utilized to correct intensity inhomogeneities within the images. Subsequent to these corrections, skull stripping was performed to remove the scalp, skull, and dura mater. Following this, tissue segmentation was carried out to isolate the gray matter, white matter, and cerebrospinal fluid. The processed images were then mapped to a prelabeled automated anatomical labeling (AAL) template, enabling the segmentation of 90 regions of interest (ROIs). Last, reconstruction of the cortical surface was accomplished. Upon completion of these procedures, metrics such as the volume of gray matter, cortical thickness, and cortical surface area for each ROI were computable (Figure 1).

### TLE morphometric feature extraction from MRI

**Figure 2:**
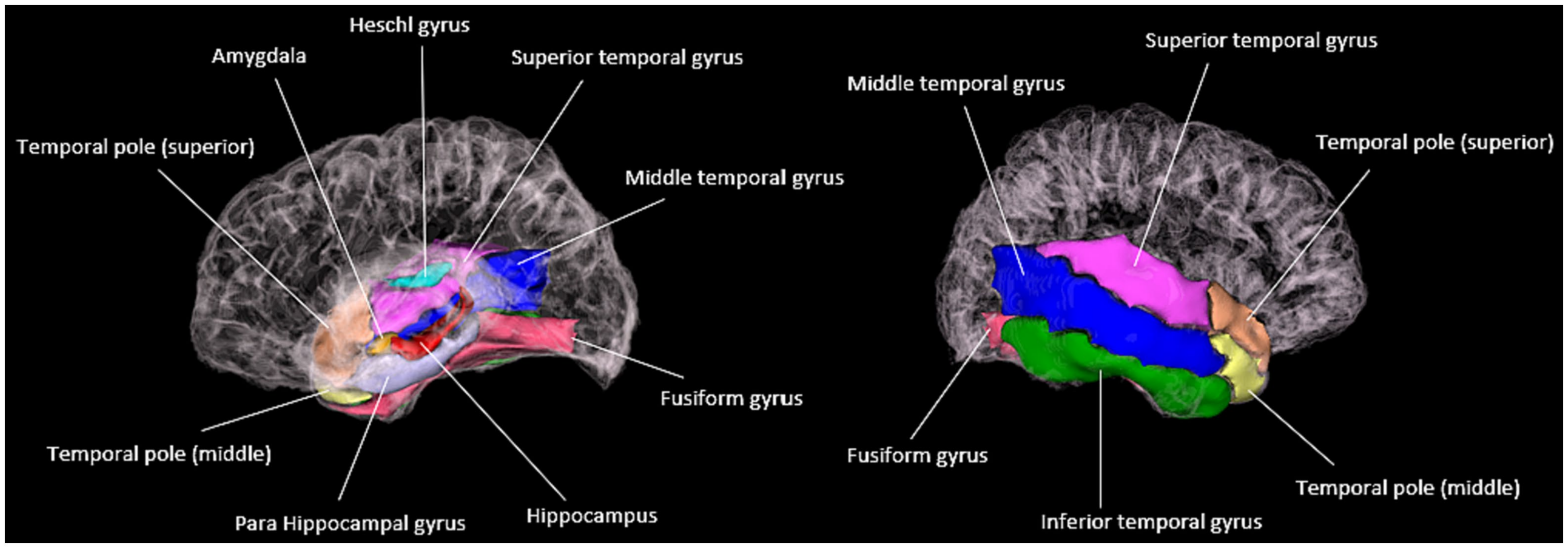
Visualization of TLE morphometric feature extraction from MR images.

Following the aforementioned image preprocessing steps, morphometric features, including the volume of gray matter, cortical thickness, and cortical surface area, can be obtained for 90 brain regions in each patient’s MRI image. After consultation with clinical experts, 10 brain regions particularly relevant to temporal lobe epilepsy (TLE) were selected for analysis. These include the hippocampus, parahippocampal gyrus, amygdala, transverse temporal gyrus, polar temporal gyrus (superior temporal gyrus), polar temporal gyrus (middle temporal gyrus), superior temporal gyrus, middle temporal gyrus, inferior temporal gyrus, and fusiform gyrus (as shown in Figure 2). Moreover, by cross-referencing the descriptions of surgical removal locations in each subject’s medical records, it was found that the typical areas excised in TLE patients were included among these 10 regions. Concurrently, the literature indicates that the temporal pole, originating amygdala, and originating hippocampal head can be identified in PET images for the localization of the onset of TLE (22). Previous research based on MRI images has confirmed a high correlation between the medial temporal lobe and TLE (23). The selection of these TLE-relevant brain region features was carried out based on this existing research and following discussion with neurosurgeons.

### Machine learning model

**Figure 3:**
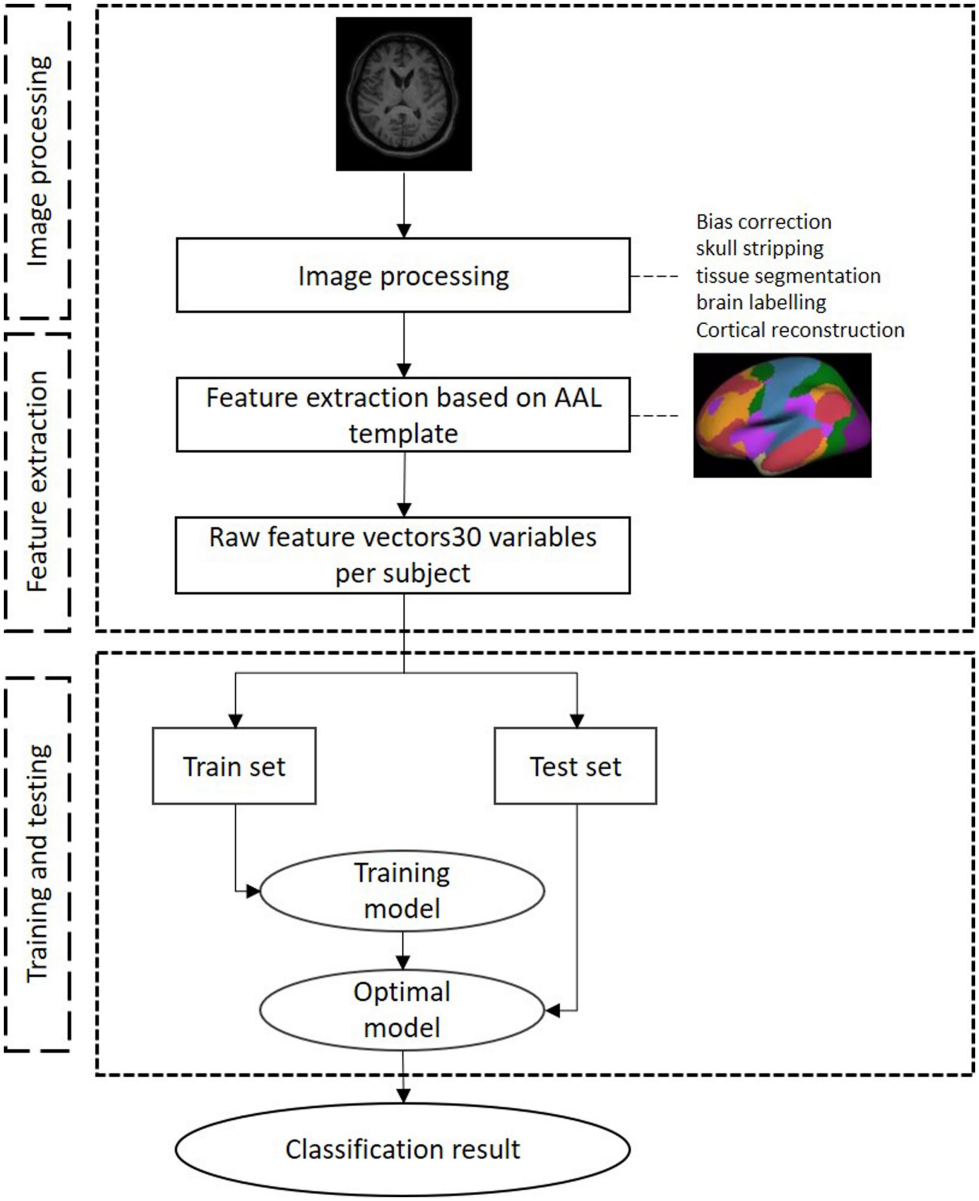
The framework of the construction of the machine learning model.

In this study, three classifiers were chosen to serve as auxiliary diagnostic models for temporal TLE, including SVM, ELM, and cmcRVFL+. SVM is a binary classification model well suited for medium- and small-sized data samples and nonlinear and high-dimensional classification problems. In this study, we selected the radial basis function (RBF) for SVM as the kernel function. ELM is a single-hidden-layer neural network model with the number of hidden nodes set to 60. It is characterized by high efficiency, accuracy, and strong generalization performance and does not require iterative learning, thus offering fast training speed. The cmcRVFL+ was an ensemble classifier (cmcRVFL+) for small sample classification (19), which combines a series RVFL as weak classifier in order to build a more robust final classifier. It formed a cascaded model that use the predict label as privileged information, which was fed into the next RVFL learner together with morphometric features. The construction of RVFL learners prevents excessive learning from the training data resulting in a less biased model. The framework for constructing these classification models is presented in Figure 3.

### Statistical analysis

All patients were randomly split into training and testing datasets. During the training phase, all training samples underwent image preprocessing and feature extraction before being fed into the classifiers for training. To address the issue of a small sample size, 5-fold cross validation was conducted for each split and the training and testing experiments were repeated five times. The average results from these experiments were used as the final outcome. The classification performance of the models was evaluated by accuracy (ACC), specificity (SPE), sensitivity (SEN), and area under the curve (AUC) values.

## Results

### Patient demographic and clinical information

The study analyzed 32 epilepsy patients, including 13 males and 19 females, ranging in age from 7 to 59 years. Fifteen of the patients had epileptic foci located in the left hemisphere, and 17 had epileptic foci in the right hemisphere. Using *t-*tests, we analyzed the age, sex, and location of the epileptic foci in these 32 patients. The results indicate that there were no significant differences between the temporal lobe epilepsy (TLE) group and the group with epileptic foci in other locations in terms of sex, age, or the lateralization of the epileptic foci. The statistically significant differences were assessed also for gray matter volume (transverse temporal gyrus with *p* = 0.005960 and superior temporal gyrus with *p* = 0.040157), cortical thickness (middle temporal gyrus with *p* = 0.032215 and fusiform gyrus with *p* = 0.022513), and cortical surface area (amygdala with *p* = 0.032215 and fusiform gyrus with *p* = 0.019514). Detailed results of the morphometric features were listed in Tables 2–4.

**Table 2:** Statistical analysis of gray matter volume features for TLE and non-TLE group in 10 temporal lobe ROIs.

	TLE	Non-TLE	*p-*value
Hippocampus	1.126985	1.004433	0.352725
Parahippocampal gyrus	1.051014	1.020202	0.373503
Amygdala	1.042755	0.973266	0.404944
Transverse temporal gyrus	1.068617	0.891413	*0.005960
Polar temporal gyrus (superior temporal gyrus)	1.038298	1.072758	0.303673
Polar temporal gyrus (middle temporal gyrus)	1.045428	0.969712	0.411268
Superior temporal gyrus	1.122513	0.919918	*0.040157
Middle temporal gyrus	1.096501	1.083007	0.361202
Inferior temporal gyrus	1.164071	0.988093	0.154616
Fusiform gyrus	1.077072	1.031041	0.388880

*p < 0.05.

**Table 3:** Statistical analysis of cortical thickness features for TLE and non-TLE group in 10 temporal lobe ROIs.

	TLE	Non-TLE	*p-*value
Hippocampus	0.974309	1.001729	0.428301
Parahippocampal gyrus	0.891543	1.008125	0.457773
Amygdala	1.069158	1.042283	0.297765
Transverse temporal gyrus	1.011662	0.983062	0.093153
Polar temporal gyrus (superior temporal gyrus)	0.882260	1.019201	0.482463
Polar temporal gyrus (middle temporal gyrus)	1.102592	0.999387	0.230359
Superior temporal gyrus	0.924386	0.985714	0.070580
Middle temporal gyrus	1.045826	0.982011	*0.032215
Inferior temporal gyrus	1.040525	0.997402	0.223118
Fusiform gyrus	0.707104	0.975444	*0.015641

*p < 0.05.

**Table 4:** Statistical analysis of cortical surface area features for TLE and non-TLE group in 10 temporal lobe ROIs.

	TLE	Non-TLE	*p* value
Hippocampus	1.108046	0.981995	0.144710
Parahippocampal gyrus	1.044484	1.030463	0.413864
Amygdala	1.213013	0.933363	*0.022513
Transverse temporal gyrus	0.993532	1.025468	0.359794
Polar temporal gyrus (superior temporal gyrus)	1.052547	1.023747	0.265886
Polar temporal gyrus (middle temporal gyrus)	1.046024	0.958219	0.144412
Superior temporal gyrus	1.017275	0.928433	0.085684
Middle temporal gyrus	1.195681	1.180977	0.465682
Inferior temporal gyrus	0.998218	0.959602	0.250236
Fusiform gyrus	0.946123	1.079781	*0.019514

*p < 0.05.

### Performance of the machine learning model

To validate the efficacy of using the ratio features of 20 selected brain regions for distinguishing between TLE and epilepsies of other localizations, three classification models were employed, including SVM, ELM, and cmcRVFL+. The classification performance was evaluated using metrics such as accuracy, sensitivity, specificity, area under the curve (AUC), and the receiver operating characteristic (ROC) curve. The performance of the three classifiers is shown in Table 5, The accuracy of the ELM classifier with gray matter volume feature reached the highest accuracy of 92.79%. The classification accuracy was higher when using gray matter volume features than when using cortical thickness or surface area features.

**Table 5:** Classification performance of the SVM, ELM and cmcRVFL+ classifiers with different features.

Classifier	Feature type	ACC%	SEN%	SPC%	AUC
SVM	Gray matter volume	86.79	97.65	95.00	0.8235
	Cortical thickness	74.29	98.23	73.00	0.7276
	Cortical surface	77.50	98.75	77.54	0.7245
ELM	Gray matter volume	92.79	93.67	93.00	0.8019
	Cortical thickness	83.07	80.67	85.33	0.7028
	Cortical surface	81.86	82.67	81.33	0.7988
cmcRVFL+	Gray matter volume	78.04	77.30	88.45	0.7667
	Cortical thickness	90.07	86.03	98.33	0.9064
	Cortical surface	79.14	82.22	72.22	0.7405

**Figure 4:**
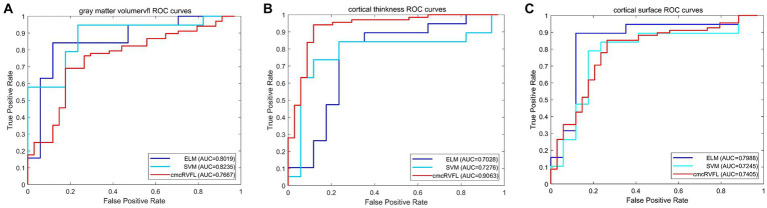
The ROC curve of the gray matter volume, cortical thickness, and cortical thickness using three different classidier. **(A)** The ROC curve using gray matter volume. **(B)** The ROC curve using cortical thickness. **(C)** The ROC curve using cortical surface area.

The ROC curves were illustrated in Figure 4. We compare the AUC value of the gray matter volume feature using SVM (AUC = 0.8235), ELM (AUC = 0.8019), and cmcRVFL+ (AUC = 0.7667). For cortical thickness, the ROC curves of SVM (AUC = 0.7276), ELM (AUC = 0.7028), and cmcRVFL+ (AUC = 0.9064) were compared. For cortical surface area, the ROC curves of SVM (AUC = 0.7245), ELM (AUC = 0.7988), and cmcRVFL+ (AUC = 0.7405) were compared. The highest AUC was obtained using cortical thickness with cmcRVFL+ classifier.

### Performance of the morphometric features

**Figure 5:**
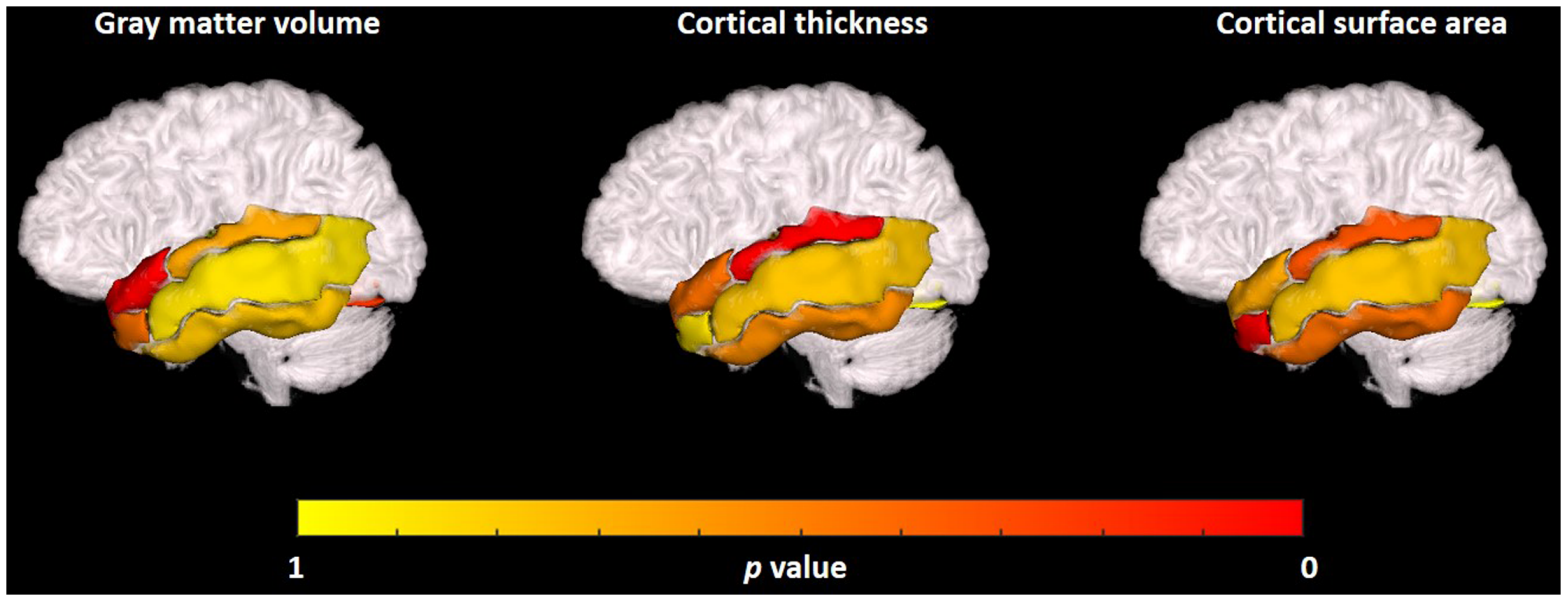
Visualization of the statistical analysis results of the gray matter volume, cortical thickness, cortical surface area in temporal related ROIs between TLE and non-TLE.

The visualization results of the statistically significant analysis of gray matter volume, cortical thickness, cortical surface area in the temporal related ROIs between TLE and non-TLE are shown in Figure 5. The color of each brain region represents the p-value of the statistical T-test for morphological features between two groups, where red indicates significant differences and yellow indicates less significant differences. The statistically significant brain regions of gray matter volume locate at transverse temporal gyrus (*p* = 0.005960) and superior temporal gyrus (*p* = 0.040157). The statistically significant brain regions of cortical thickness locate at middle temporal gyrus (*p* = 0.032215) and fusiform gyrus (*p* = 0.015641). The statistically significant brain regions of cortical surface area locate at amygdala (*p* = 0.022513) and fusiform gyrus (*p* = 0.019514). Details of the statistical analysis results of gray matter volume, cortical thickness, cortical surface area are shown in Tables 2–4.

## Discussion

### Machine learning model for TLE detection

Some patients with temporal lobe epilepsy (TLE) exhibit no discernible lesions on MRI, termed MRI-negative TLE. The diagnosis of TLE relies on imaging studies, requiring the exclusion of hippocampal sclerosis and other structural anomalies. Previous research typically utilized 1.5 T MRI scans for case selection, which risks overlooking subtle lesions and consequently misclassifying them as MRI-negative patients. The widespread use of 3.0 T high-resolution cranial MRI in recent years has increased the detection rate of epileptogenic foci in TLE patients by approximately 20–48%. While some MRI-negative cases can be accurately diagnosed with a structural abnormality using 3.0 T MRI, 20–30% of TLE patients still do not show any obvious lesions on MRI and are considered to be strictly MRI-negative TLE. The debate continues as to whether TLE is a distinct spectrum of the disease; some scholars suggest the possible presence of minor hippocampal sclerosis, visible only on a histopathological level. Studies have found that certain brain regions in TLE patients show a reduced volume of white matter compared to the control group, while other studies indicate atrophy in the ipsilateral entorhinal cortex in MRI-negative TLE patients. Therefore, the clinical and morphometric characteristics of TLE remain ambiguous.

To address these challenges, this study proposes a TLE identification method based on morphological features of temporal lobe regions and machine learning. Three machine learning models, namely, SVM, ELM, and cmcRVFL+ were employed for classification experiments. SVM is a supervised learning model commonly used for pattern recognition, classification, and regression analysis. It is well suited for binary classification tasks involving medium-to-small size datasets, nonlinearity, and high dimensionality. Compared with SVM, the ELM consists of a simple three-layer architecture comprising input, hidden, and output layers, forming a single-hidden-layer feedforward neural network. It only requires setting the number of hidden layer nodes, obviating the need to adjust the input weights and thresholds of the hidden layer, eventually producing an optimal solution. ELM thus demonstrates better adaptability using gray matter volume (ACC = 92.79%, AUC = 0.8019). The cmcRVFL+ is an ensemble classifier for small sample classification, which combines a series RVFL as weak classifier in order to build a more robust final classifier. It formed a cascaded model that use the predict label as privileged information, which was fed into the next RVFL learner together with morphometric features. The construction of RVFL learners prevents excessive learning from the training data resulting in a less biased model. The classification performance of cmcRVFL+ were improved (ACC = 90.07%, AUC = 0.9064) using cortical thickness. All these classification models were used to evaluated the effectiveness of morphometric features for MRI-negative TLE detection, the classifier based on ELM outperformed in distinguishing TLE from epilepsies using temporal lobe gray matter volume features.

### Analysis of gray matter volume alterations in the temporal lobe ROIs of TLE patients

The t test method was utilized to analyze the statistical significance of volumetric features of gray matter and cortical surface area in the bilateral brain regions of three clinically TLE-relevant areas: the hippocampus, the transverse temporal gyrus, and the middle temporal gyrus. As indicated in Table 4, the gray matter volume features in the bilateral regions of the hippocampus showed a significant difference with a *p* value of 0.0086. Additionally, the gray matter volume features in the transverse temporal gyrus displayed a significant difference between the TLE group and the group with epilepsies originating in other brain regions, with a *p*-value of 0.045. Ultimately, we selected the ratio of three features in the bilateral brain regions of 10 areas within the surgical zone as features for machine learning classification, and this yielded the most effective results.

Recent research indicates that subtle structural changes in subregions of the hippocampus could lead to neurological and psychiatric disorders, including epilepsy, Alzheimer’s disease, major depressive disorder, posttraumatic stress disorder, and schizophrenia (24). Temporal lobe epilepsy with hippocampal sclerosis (TLE-HS) is a common subtype of temporal lobe epilepsy and a classical focal epilepsy syndrome. Epileptic seizures in these patients generally originate from the medial temporal lobe or simultaneously involve limbic structures and are accompanied by hippocampal sclerosis (25). For patients with temporal lobe epilepsy where the hippocampus is the frequent epileptogenic zone, 75% ultimately progress to drug-resistant epilepsy. The seizure types in TLE-HS patients are diverse, with causes ranging from perinatal hypoxia-ischemia-induced brain damage, brain malformations, cerebral vascular malformations, trauma, and infection-induced scar tissue, leading to hippocampal degenerative sclerosis and forming a new epileptogenic focus. Reports indicate that hippocampal sclerosis is also a significant cause of epilepsy, ultimately resulting in a “dual-source” epileptogenic focus (26).

Studies have found that MRI-negative TLE patients exhibit atrophy in the transverse temporal gyrus area. The possible mechanism involves the reduction of output neurons in the epileptogenic zone, resulting in diminished afferent input and consequently reduced volume in brain regions that are synaptically connected to the epileptogenic zone (27). Epileptic discharges are not confined to the epileptogenic focus but propagate widely to other brain areas. Local neurotoxins can cause damage to neurons not only in the epileptogenic zone but also in remote areas, leading to neuronal loss. Furthermore, there may be subtle cortical developmental anomalies and other pathological changes in brain tissue. The collective impact of these multiple factors is likely the pathophysiological mechanism underlying the reduction in gray matter volume seen in epilepsy patients (28).

### Limitation

Despite achieving promising research results, this study has several limitations: (1) The small sample size is a main concern for overfitting. We have taken actions to deal with this issue that an ensemble classifier was used. The cmcRVFL+ combines multiple RVFL classifiers to build a more robust classifier. It formed a cascaded model that use the predict label as privileged information, which was fed into the next RVFL learner together with features. The construction of RVFL learners prevents excessive learning from the training data resulting in a less biased model. The overfitting issue was further ensured by the use of 5-fold cross validation and repeated experiment. Future studies will include additional samples for validation, addressing the overfitting limitations. (2) We only used imaging data from a single modality. If data from multiple modalities were available, it would be possible to extract features from more dimensions for more accurate classification. Future experiments could be conducted using data from multiple modalities, and other types of information will be used to improve the model’s classification accuracy, such as neuropsychological assessments.

## Conclusion

In this paper, we addressed the problem of low identification rates of MRI-negative TLE in clinical settings by using machine learning methods. We classified patients based on three types of features within 20 bilateral brain regions associated with the temporal lobe, as identified by the automated anatomical labeling (AAL) template within the surgical resection area. The results showed that the use of an extreme learning machine (ELM) classifier, combined with features such as cortical thickness in specific brain regions, yielded good results in identifying temporal lobe epilepsy as distinct from epilepsy originating from other brain areas.

## Data availability statement

The data that support the findings of this study are available from the corresponding author, daiyk@sibet.ac.cn, upon reasonable request.

## Ethics statement

The studies involving humans were approved by Ethics Committee of the First Affiliated Hospital of Soochow University. The studies were conducted in accordance with the local legislation and institutional requirements. Written informed consent for participation in this study was provided by the participants’ legal guardians/next of kin.

## Author contributions

LY: Conceptualization, Methodology, Writing – original draft. BP: Writing – original draft, Methodology. WG: Writing – review & editing. RA: Software, Visualization, Writing – review & editing. YL: Validation, Writing – review & editing. JL: Project administration, Writing – review & editing. MZ: Resources, Writing – review & editing. HH: Data curation, Writing – review & editing. ZL: Investigation, Writing – review & editing. CP: Formal analysis, Writing – review & editing. YD: Funding acquisition, Supervision, Writing – review & editing. YS: Funding acquisition, Supervision, Writing – review & editing.

## References

[1] Fisher RS Acevedo C Arzimanoglou A Bogacz A Cross JH Elger CE. ILAE official report: a practical clinical definition of epilepsy. Epilepsia. (2014) 55:475–82. doi: 10.1111/epi.12550 24730690

[2] Fisher RS Boas W v E Blume W Elger C Genton P Lee P. Epileptic seizures and epilepsy: definitions proposed by the international league against epilepsy (ILAE) and the International Bureau for Epilepsy (IBE). Epilepsia. (2005) 46:470–2. doi: 10.1111/j.0013-9580.2005.66104.x, PMID: 15816939

[3] Arle JE Perrine K Devinsky O Doyle WK. Neural network analysis of preoperative variables and outcome in epilepsy surgery. J Neurosurg. (1999) 90:998–1004. doi: 10.3171/jns.1999.90.6.0998, PMID: 10350243

[4] Téllez-Zenteno JF Hernández-Ronquillo L. A review of the epidemiology of temporal lobe epilepsy. Epilepsy Res Treatment. (2012) 2012:1–5. doi: 10.1155/2012/630853, PMID: 22957234 PMC3420432

[5] Stefan H Lopes da Silva FH. Epileptic neuronal networks: methods of identification and clinical relevance. Front Neurol. (2013) 4:8. doi: 10.3389/fneur.2013.00008 23532203 PMC3607195

[6] Samanta D Singh R Gedela S Scott Perry M Arya R. Underutilization of epilepsy surgery: part II: strategies to overcome barriers. Epilepsy Behav. (2021) 117:107853. doi: 10.1016/j.yebeh.2021.107853, PMID: 33678576 PMC8035223

[7] Hermann BP Struck AF Busch RM Reyes A Kaestner E McDonald CR. Neurobehavioural comorbidities of epilepsy: towards a network-based precision taxonomy. Nat Rev Neurol. (2021) 17:731–46. doi: 10.1038/s41582-021-00555-z, PMID: 34552218 PMC8900353

[8] Krakow K Wieshmann UC Woermann FG Symms MR McLean MA Lemieux L. Multimodal MR imaging: functional, diffusion tensor, and chemical shift imaging in a patient with localization-related epilepsy. Epilepsia. (1999) 40:1459–62. doi: 10.1111/j.1528-1157.1999.tb02021.x, PMID: 10528945

[9] Zubler F Seeck M Landis T Henry F Lazeyras F. Contralateral medial temporal lobe damage in right but not left temporal lobe epilepsy: a 1H magnetic resonance spectroscopy study. J Neurol Neurosurg Psychiatry. (2003) 74:1240–4. doi: 10.1136/jnnp.74.9.1240, PMID: 12933926 PMC1738688

[10] Helmstaedter C Petzold I Bien CG. The cognitive consequence of resecting nonlesional tissues in epilepsy surgery—results from MRI-and histopathology-negative patients with temporal lobe epilepsy. Epilepsia. (2011) 52:1402–8. doi: 10.1111/j.1528-1167.2011.03157.x, PMID: 21740419

[11] McDonald CR Hagler DJ Jr Ahmadi ME Tecoma E Iragui V Gharapetian L. Regional neocortical thinning in mesial temporal lobe epilepsy. Epilepsia. (2008) 49:794–803. doi: 10.1111/j.1528-1167.2008.01539.x, PMID: 18266751

[12] Singh P Kaur R Saggar K Singh G Aggarwal S. Amygdala volumetry in patients with temporal lobe epilepsy and normal magnetic resonance imaging. Pol J Radiol. (2016) 81:212–8. doi: 10.12659/PJR.896077, PMID: 27231493 PMC4865273

[13] Blümcke I Thom M Aronica E Armstrong DD Bartolomei F Bernasconi A. International consensus classification of hippocampal sclerosis in temporal lobe epilepsy: a task force report from the ILAE commission on diagnostic methods. Epilepsia. (2013) 54:1315–29. doi: 10.1111/epi.12220, PMID: 23692496

[14] Lampinen B Zampeli A Björkman-Burtscher IM Szczepankiewicz F Källén K Compagno Strandberg M. Tensor-valued diffusion MRI differentiates cortex and white matter in malformations of cortical development associated with epilepsy. Epilepsia. (2020) 61:1701–13. doi: 10.1111/epi.16605, PMID: 32667688 PMC7963222

[15] Huang S Cai N Pacheco PP Narrandes S Wang Y Xu W. Applications of support vector machine (SVM) learning in cancer genomics. Cancer Genomics Proteomics. (2018) 15:41–51. doi: 10.21873/cgp.20063 PMID: 29275361 PMC5822181

[16] Huang C Zhou Y Zhong Y Wang X Zhang Y. The bilateral precuneus as a potential neuroimaging biomarker for right temporal lobe epilepsy: a support vector machine analysis. Front Psych. (2022) 13:923583. doi: 10.3389/fpsyt.2022.923583, PMID: 35782449 PMC9240203

[17] Chorowski J Wang J Zurada JM. Review and performance comparison of SVM-and ELM-based classifiers[J]. Neurocomputing. (2014) 128:507–16. doi: 10.1016/j.neucom.2013.08.009

[18] Wang Y Li Z Feng L Zheng C Zhang W. Automatic detection of epilepsy and seizure using multiclass sparse extreme learning machine classification. Comput Math Methods Med. (2017) 2017:1–10. doi: 10.1155/2017/6849360, PMID: 28706561 PMC5494790

[19] Shi J Xue Z Dai Y Peng B Dong Y Zhang Q. Cascaded multi-column RVFL+ classifier for single-modal neuroimaging-based diagnosis of Parkinson’s[J]. IEEE Trans Biomed Eng. (2018) 66:2362–71. doi: 10.1109/TBME.2018.2889398, PMID: 30582522

[20] Goel T Sharma R Tanveer M Suganthan PN Maji K Pilli R. Multimodal neuroimaging based Alzheimer’s disease diagnosis using evolutionary RVFL classifier. IEEE J Biomed Health Inform. (2023):1–9. doi: 10.1109/JBHI.2023.3242354 37022418

[21] Dai Y Wang Y Wang L Wu G Shi F Shen D. aBEAT: a toolbox for consistent analysis of longitudinal adult brain MRI. PLoS One. (2013) 8:e60344. doi: 10.1371/journal.pone.0060344, PMID: 23577105 PMC3616755

[22] Tan Y-L Kim H Lee S Tihan T ver Hoef L Mueller SG. Quantitative surface analysis of combined MRI and PET enhances detection of focal cortical dysplasias. NeuroImage. (2018) 166:10–8. doi: 10.1016/j.neuroimage.2017.10.065, PMID: 29097316 PMC5748006

[23] Hu W-h Wang X Liu L-n Shao X-q Zhang K Ma Y-s. Multimodality image postprocessing in detection of extratemporal MRI-negative cortical dysplasia. Front Neurol. (2018) 9:450. doi: 10.3389/fneur.2018.00450, PMID: 29963006 PMC6010529

[24] Coras R Pauli E Li J Schwarz M Rössler K Buchfelder M. Differential influence of hippocampal subfields to memory formation: insights from patients with temporal lobe epilepsy. Brain. (2014) 137:1945–57. doi: 10.1093/brain/awu100, PMID: 24817139

[25] Royer S Sirota A Patel J Buzsáki G. Distinct representations and theta dynamics in dorsal and ventral hippocampus. J Neurosci. (2010) 30:1777–87. doi: 10.1523/JNEUROSCI.4681-09.2010, PMID: 20130187 PMC2825159

[26] Mueller SG Laxer KD Schuff N Weiner MW. Voxel-based T2 relaxation rate measurements in temporal lobe epilepsy (TLE) with and without mesial temporal sclerosis. Epilepsia. (2007) 48:220–8. doi: 10.1111/j.1528-1167.2006.00916.x, PMID: 17295614 PMC2744642

[27] Bruggemann JM Wilke M Som SS Bye AME Bleasel A Lawson JA. Voxel-based morphometry in the detection of dysplasia and neoplasia in childhood epilepsy: limitations of grey matter analysis. J Clin Neurosci. (2009) 16:780–5. doi: 10.1016/j.jocn.2008.08.025 19303304

[28] Bonilha L Rorden C Castellano G Pereira F Rio PA Cendes F. Voxel-based morphometry reveals gray matter network atrophy in refractory medial temporal lobe epilepsy. Arch Neurol. (2004) 61:1379–84. doi: 10.1001/archneur.61.9.1379, PMID: 15364683

